# Predicting subway passenger flows under different traffic conditions

**DOI:** 10.1371/journal.pone.0202707

**Published:** 2018-08-27

**Authors:** Ximan Ling, Zhiren Huang, Chengcheng Wang, Fan Zhang, Pu Wang

**Affiliations:** 1 School of Traffic and Transportation Engineering, Central South University, Changsha, Hunan, China; 2 Shenzhen Institutes of Advanced Technology, Chinese Academy of Sciences, Shenzhen, Guangdong, China; Beihang University, CHINA

## Abstract

Passenger flow prediction is important for the operation, management, efficiency, and reliability of urban rail transit (subway) system. Here, we employ the large-scale subway smartcard data of Shenzhen, a major city of China, to predict dynamical passenger flows in the subway network. Four classical predictive models: historical average model, multilayer perceptron neural network model, support vector regression model, and gradient boosted regression trees model, were analyzed. Ordinary and anomalous traffic conditions were identified for each subway station by using the density-based spatial clustering of applications with noise (DBSCAN) algorithm. The prediction accuracy of each predictive model was analyzed under ordinary and anomalous traffic conditions to explore the high-performance condition (ordinary traffic condition or anomalous traffic condition) of different predictive models. In addition, we studied how long in advance that passenger flows can be accurately predicted by each predictive model. Our finding highlights the importance of selecting proper models to improve the accuracy of passenger flow prediction, and that inherent patterns of passenger flows are more prominently influencing the accuracy of prediction.

## Introduction

Public transportation plays an indispensable role in modern big cities. Developing public transportation is regarded as the most effective way to solve the ubiquitous traffic congestion problems [1, 2]. The subway is regarded as the backbone of urban public transportation, and is characterized by high speed, convenience, and mass flow features [3–7]. Despite the fact that subway services have been continuously improved in many big cities, the upgraded supply usually cannot meet the even faster growing demands of human mobility, especially in developing countries. Compared with opening new lines or increasing the operating frequency of trains, intelligent operation is a smarter and more cost-efficient way to improve the level of service. This calls for accurate and robust prediction of passenger flows to guide better use of the capacity of subway networks. Despite that some passenger flow prediction models have been proposed, we revisited this important problem from two new perspectives.

First, we analyzed the performance of different predictive models under different passenger flow (traffic) conditions. In general, traffic conditions can be classified into ordinary conditions, for example morning commutes in a typical weekday, and anomaly conditions, such as bursts of passenger flow in a specific subway station due to a large commercial or recreational event. Moreover, the variance of travel time under the congestion state is remarkably larger than that under the free-flow state [8]. We identified the traffic conditions of each subway station using the density-based spatial clustering of applications with noise (DBSCAN) algorithm, and explored the high-performance passenger flow models under different traffic conditions.

Second, previous works rarely explored how long in advance that passenger flows can be well predicted by each kind of predictive model. Most models were tested by inputting data collected in a time window to predict passenger flows in the next adjacent time window. However, this type of input data setting is hard to implement in practice because collection of smartcard data usually has a delay. Moreover, for some practical applications, such as preventing a large crowd gathering that may cause a dangerous crowding situation, it is important to predict passenger flows a long time before high-density crowding is realized because it is difficult to evacuate high-density crowds both safely and rapidly.

In the following, we make a brief review of existing traffic prediction models, which can be generally classified into three types: (1) mathematical analytical models; (2) traffic simulation models; and (3) knowledge discovery models.

Early traffic prediction models were mostly based on mathematical analytic approaches. Time series models, which include the auto regression (AR) model, moving average (MA) model, auto regressive moving average (ARMA) model, and autoregressive integrated moving average (ARIMA) model, are typical examples. In 1927, Yule developed the AR model to study the periodicities of Wolfer’s sunspot numbers [9]. In this AR model, the curve of the time series was fit by the linear combination of the observed historical values. Walker developed the MA model based on the AR model in 1931 [10]. The MA model used a linear combination of historical random disturbances and prediction errors to obtain the current predictive value. In the same year, Walker proposed the ARMA model, which combined the AR model and MA model. In 1970, Box and Jenkins proposed the ARIMA model [11], which incorporated a differencing process (data values were replaced by the differences of current data values and historical data values) in the ARMA model.

Despite the long history of the time series model, it was first used in transportation studies by Ahmed and Cook in 1979 [12]. They employed the ARIMA model to predict traffic flow in freeways; however, the accuracy of prediction was not satisfying. In the 1980s, Stephanedes and Okutani respectively applied the historical average (HA) model and the Kalman filter model to the urban traffic control system of Minneapolis-St and Nagoya City [13, 14]. Recently, Wang *et al*. [15] developed a general approach for real-time freeway traffic state prediction based on stochastic macroscopic traffic flow modeling and extended Kalman filtering, and Li studied the prediction of traffic flow based on interval type-2 fuzzy sets theory [16]. Given that the HA model is prominently influenced by random disturbance, the Kalman filter model was used to adjust the Kalman gain weight every time, resulting in a heavy computing burden. Time series of traffic states sometimes show obvious periodic variation (quarterly, monthly, weekly, etc.), and thus, the seasonal ARIMA (SARIMA) model was developed to capture periodic variations of traffic states by Williams and Hoel in 2003 [17]. They applied the SARIMA model in the prediction of traffic flow in freeways, and found that it outperformed the HA model. Recently, Schimbinschi *et al*. [18] proposed a novel model named topology-regularized universal vector autoregression (TRU-VAR) for traffic flow prediction, which performs better than ARIMA model. In addition, Xue *et al*. [19] proposed a hybrid model combining the time series model with interactive multiple model (IMM) algorithm to predict the short-term bus passenger demand, it is superior to times series model. Ma *et al*. [20] used a geographically and temporally weighted regression (GTWR) model to identify the spatiotemporal influence of the built environment on transit ridership.

Traffic simulation models were widely used with the popularization of computers in scientific research. In 2001, Chrobok *et al*. [21] presented an approach based on a micro-simulator to predict traffic flow in the freeway network of North Rhine-Westphalia. In 2010, McCrea *et al*. [22] proposed a novel hybrid approach that combines the advantages of the traffic simulation model and linear system theory. In their model, traffic dynamics was first simulated using a continuum mathematical model to obtain relevant traffic parameters of road segments, and the obtained parameters were used as inputs for the Bayesian model for traffic flow prediction. Under the same requirement of prediction accuracy, the hybrid approach improved the computing efficiency compared to the Bayesian network model.

In recent years, knowledge discovery methods have been used more frequently in traffic prediction. Representative methods include nonparametric regression analysis, artificial neural networks, support vector machines, wavelet analysis, and gradient boosting decision tree [23]. In 1991, Davis and Nihan applied nonparametric regression to predict traffic flow in a freeway; however, the accuracy of prediction was lower than that of the linear time-series method [24]. Twelve years later, Clark applied the method of multivariate nonparametric regression to predict the traffic state of a motorway [25]. The method was simple and easy to implement, requiring only modest data storage, and produced reasonably accurate short-term forecasts of traffic flow and loop occupancies (in the percentage of time a loop is covered by a vehicle).

Artificial neural networks were born in the 1940s, and first introduced in traffic flow prediction by Vythoulkas in 1993 [26]. He employed an artificial neural network to predict the traffic state of a city road network. Two years later, Dougherty summarized the application of neural networks in transportation studies [27]. The transportation research community saw an explosion of interest on neural networks in the 1990s. A variety of neural network models have been proposed to predict traffic conditions. Representative examples include the multilayer perceptron neural network model [28], radial basis function neural network [29, 30], spectral basis artificial neural network [31], time delayed neural network [32], and recurrent neural network [33]. Models combining neural networks with other factors (e.g., time series [34], genetic algorithms [35], fuzzy logic rules [36], empirical mode decomposition [37], etc.) were also studied.

Support vector machines were formally published in 1995 [38], and studies on support vector regression (SVR) began in 1997 [39]. Support vector regression was used for travel-time prediction [40, 41]. Wu *et al*. [40] validated the feasibility of applying support vector regression in travel-time prediction, the mean relative errors for traveling different distances were less than 5% in the test dataset. Vanajakshi *et al*. [41] found the support vector regression performs better than artificial neural network when the training data is less or when there are a lot of variations in the training data. Recently, Jiang *et al*. [42] combined the ensemble empirical mode decomposition with gray support vector machine to predict the short-term passenger flow of high-speed rail (HSR), and the mean absolute percentage errors of the hybrid model is about 6%, which performs better than the SVM model and the ARIMA model.

Wavelet analysis, which was developed in the 1980s, is usually used to decompose a set of original traffic flow signals into signals with different time series to reflect and distinguish the internal variation trend and stochastic disturbance of traffic flows. He *et al*. [43] proposed a method based on wavelet decomposition and reconstruction combined with the time series model for traffic volume prediction. And the processed signals with different characteristics can be combined with the dynamic neural network [44], support vector machines [45], and other methods, to predict traffic flow.

In this study, the smartcard data of more than 6 million subway passengers and geographic information data of the Shenzhen subway network were used. We analyzed four classical predictive models: the historical average (HA) model, multilayer perceptron (MLP) neural network model, support vector regression (SVR) model, and gradient boosted regression trees (GBRT) model. Different from previous studies, we explored the high-performance models under different traffic conditions, and studied how long in advance that passenger flows could be accurately predicted by each predictive model.

The paper is organized as follows. Section II describes the geographic information data and passenger mobility data used in this study. Section III introduces the passenger flow prediction models and algorithm used to classify passenger flow (traffic) conditions. Section IV analyzes and discusses the passenger flow prediction results of different models, and identifies the high-performance models under different traffic conditions and different model implementation conditions (how long passenger flows are predicted in advance). Section V concludes the results, and discusses future research directions.

## Materials and methods

### Data

The geographic information systems (GIS) data and smartcard data of Shenzhen subway passengers were both provided by Shenzhen Transportation Authority. Data collection was conducted in 2014; the collection of smartcard data was from October 1, 2014 to December 31, 2014. In 2014, the subway network consisted of 118 subway stations. Stations opened after 2014 were not considered due to lack of smartcard data for the new stations. Once a subway passenger employs his/her smartcard when entering or existing a subway station, the time, card ID, and subway station ID are recorded. In the three-month data collection period, a total of 262 million passenger records were generated. For some days, there was data missing for a few hours or the whole day; therefore, only days with complete records were used in this study (80 days in total).

**Fig 1:**
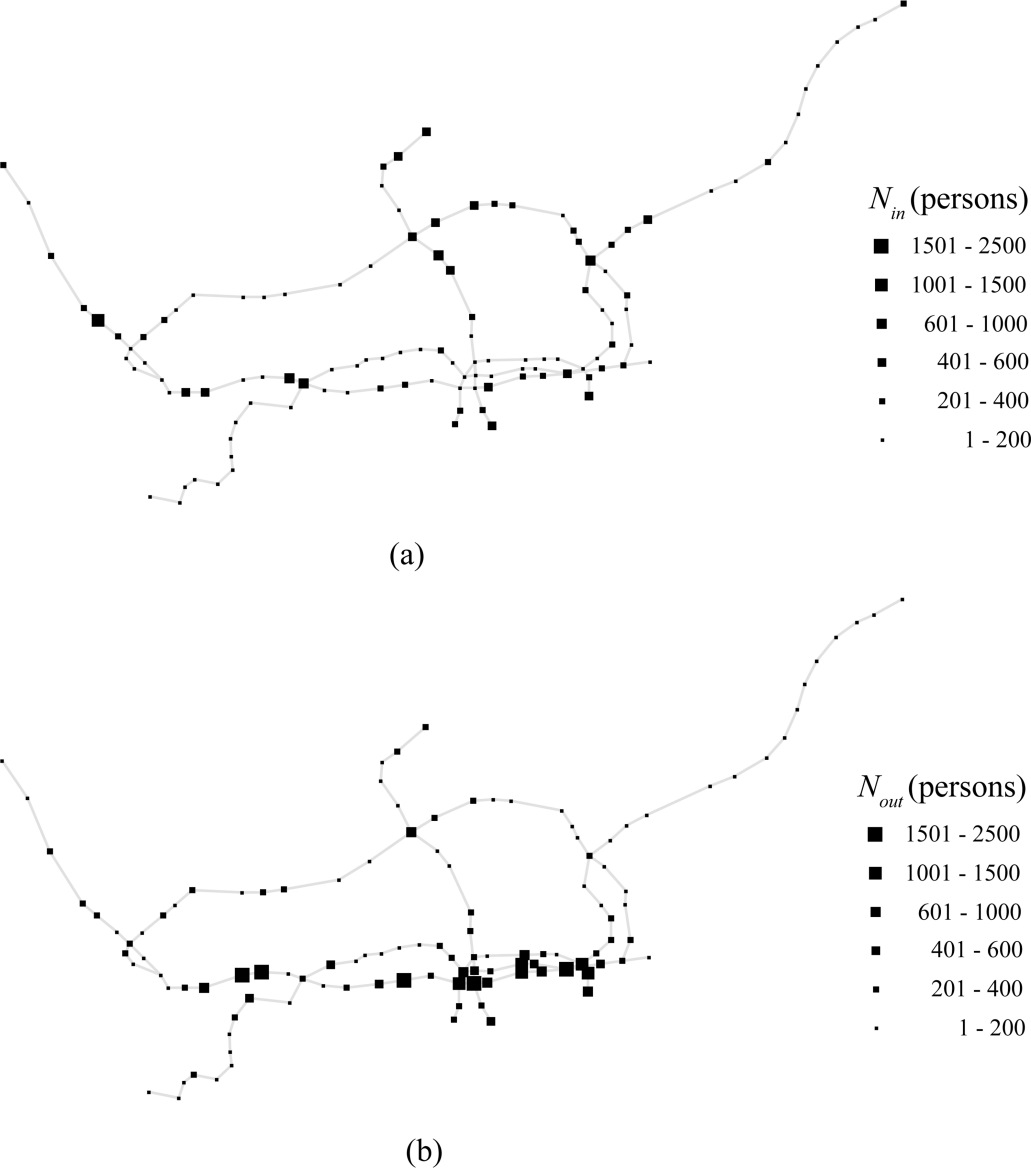
(a), (b) In-passenger-flow *N*_*in*_(*s*,*t*) and out-passenger-flow *N*_*out*_(*s*,*t*) of each subway station *s* during the time window 9:00 a.m.–9:15 a.m. of a typical weekday in 2014.

**Fig 2:**
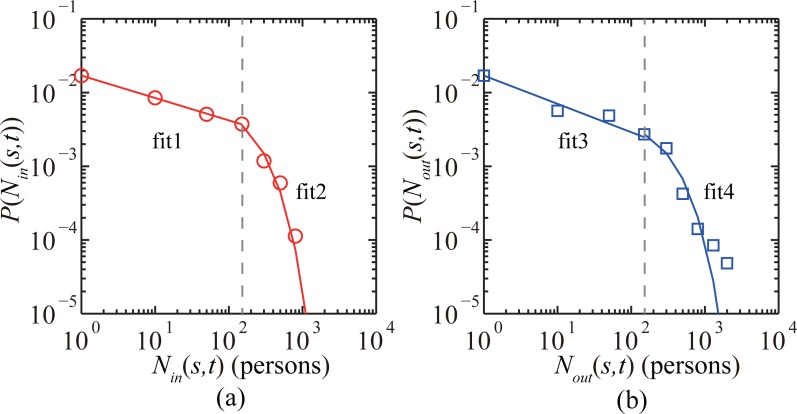
Distributions of passenger-flow show heterogenous patterns in different subway stations. (a) is for in-passenger-flow *N*_*in*_(*s*,*t*), (b) is for out-passenger-flow *N*_*out*_(*s*,*t*).

The three-month observation period was split into 7,680 time windows, with each time window spanning 15 min. Taking the operation period of Shenzhen Metro into consideration, the time period of data collection for each day was from 7:00 a.m. to 10:30 p.m.. Therefore, there are only 62 time windows in each day used for training data and testing data. The time windows from 10:30 p.m. to 7:00 a.m. are not considered because few smartcard data are available during the late-night period. We calculated the number of passengers entering a subway station *s* during each time window *t*, in-passenger-flow *N*_*in*_(*s*,*t*), and the number of passengers exiting a subway station *s* during each time window *t*, out-passenger-flow *N*_*out*_(*s*,*t*) (Fig 1A and 1B). Heterogeneous distribution of passenger flows is observed in the studied subway network (Fig 2A and 2B). The in-passenger-flow can be approximated by two different fitting functions for large and small *N*_*in*_(*s*,*t*) (gray dashed lines are plotted to guide the eyes):

fit1: *P*(*N*_*in*_(*s*,*t*)) = 0.017 (*N*_*in*_(*s*,*t*))^−0.304^ when *N*_*in*_(*s*,*t*) ≤ 150 persons;
fit2: *P*(*N*_*in*_(*s*,*t*)) = 0.009 exp(−0.006 *N*_*in*_(*s*,*t*)) when *N*_*in*_(*s*,*t*) > 150 persons.

The out-passenger-flow also can be approximated by two different fitting functions for large and small *N*_*out*_(*s*,*t*) (gray dashed lines are plotted to guide the eyes):

fit3: *P*(*N*_*out*_(*s*,*t*)) = 0.017 (*N*_*out*_(*s*,*t*))^−0.384^ when *N*_*out*_(*s*,*t*) ≤ 150 persons;
fit4: *P*(*N*_*out*_(*s*,*t*)) = 0.005 exp(−0.004 *N*_*out*_(*s*,*t*)) when *N*_*out*_(*s*,*t*) > 150 persons.

Roughly 58.47% of in-passenger-flow *N*_*in*_(*s*,*t*) and 50% of out-passenger-flows *N*_*out*_(*s*,*t*) were smaller than 200 passengers/15 min; for some stations, passenger flows were larger than 1,000 passengers/15 min. In the following sections, measured in-passenger-flow *N*_*in*_(*s*,*t*) and out-passenger-flow *N*_*out*_(*s*,*t*) were used as the ground truth data to train the passenger flow prediction models and validate the predictive results.

The used subway smartcard data were split into two parts. The first part of the data, which recorded the subway passenger trips generated during October and November of 2014, were used as the training dataset. The second part of the data, which recorded the subway passenger trips generated during December of 2014, were used as the testing dataset. Training datasets were denoted by *D* = {(***x***_1_,***y***_1_),(***x***_2_,***y***_2_),…,(***x***_*n*_,***y***_*n*_)}, where ***x***_*n*_ ∈ *R*^*d*^ represent the input features of the training data, and ***y***_*n*_ ∈ *R*^*l*^ represent the output results of the training data. The sample size *n* equals 59 because there were 59 days’ smartcard data in the training dataset. Data dimensions *d* and *l* represent the number of input and output features used in the models respectively.

### The prediction models

When predicting the passenger flow of a subway station *s* during a time window *t*_*target*_, subway station *s* is called target station, and time window *t*_*target*_ is called target time window. We evaluated the performances of four predictive models under different model implementation conditions; predictions were made in different number (*n*_*step*_) of time windows before *t*_*target*_, and *n*_*step*_ =1,2,…7,8 were tested. Here, we briefly introduce the advantageous and disadvantageous features of each of the four predictive models used in this study. The HA model is easy to implement in practice, but performs poorly under unexpected traffic conditions. The multilayer perceptron (MLP) neural network employed in study is trained using back-propagation. In general, the MLP model works well in capturing complex and nonlinear relations; however, it usually requires a large volume of data and complex training procedures. For the employed SVR model, a linear kernel function was used to predicts passenger flows; however, the selection of best kernel functions is an unsolved problem in this scientific community. Lastly, the GBRT model uses a negative gradient of loss function as an estimate of residuals. In general, the GBRT model also works well in exploring complex and nonlinear relations; however, it cannot train data parallelly.

In the generated HA model, the average in-passenger-flow (or average out-passenger-flow) during the target time window *t*_*target*_ of all days in the training dataset were used as the predictive result in the target time window for all days in the testing dataset. Clearly, the HA model was unable to capture the random disturbances of passenger flows, and therefore had the worst prediction accuracy and served as a baseline model for comparison with the other three models. For the MLP model, SVR model, and GBRT model, in-passenger-flows *N*_*in*_(*s*,*t*) during time window *t* of all days in the training dataset were used as inputs, and in-passenger-flows *N*_*in*_(*s*,*t*_*target*_) during the target time window of all days in the training dataset were used as outputs to train the predictive model; *t* is *n*_*step*_ time windows before the target time window *t*_*target*_. In a given day of the testing dataset, the in-passenger-flows *N*_*in*_(*s*,*t*) were used as inputs to predict *N*_*in*_(*s*,*t*_*target*_), where *t* is *n*_*step*_ time windows before the target time window *t*_*target*_. Parameter *n*_*step*_ determines how long in advance predictions are conducted. Similarly, models were generated to predict *N*_*out*_(*s*,*t*_*target*_). Methods for generating the MLP model, SVR model, and GBRT model are briefly described in the following subsections. Please refer to the literature [46–49] for further details on the generations of these models.

The training dataset *D* = {(***x***_1_,***y***_1_),(***x***_2_,***y***_2_),…,(***x***_*n*_,***y***_*n*_)}, ***x***_*n*_ ∈ *R*^*d*^,***y***_*n*_ ∈ *R*^*l*^ were used in the MLP model, SVR model, and GBRT model. Parameters *d* and *l* represent the dimensions of ***x*** and ***y***, respectively. In this paper, parameters *d* = 1, *l* = 1 are selected because only passenger flows of a station itself are used as the model inputs to predict the passenger flows of the station. Parameter *n* represents the sample size of *D* (i.e., 59 days’ smartcard data in the training dataset).

Taking the prediction of out-passenger-flows *N*_*out*_(*s*,*t*_*target*_) at the subway station “Window of World” during 9:00 a.m.–9:15 a.m. of December 30 as an example, *s* denotes the “Window of World” subway station, and *t*_*target*_ denotes the target time window 9:00 a.m.–9:15 a.m. When predicting passenger flows at one time window ahead of the target time window (*n*_*step*_ = 1), historical passenger flows at the station *s* during the time window *t*_*target*_ − 1 of all days in the training dataset *D* = {(***x***_1_,***y***_1_),(***x***_2_,***y***_2_),…,(***x***_*n*_,***y***_*n*_)} are used. For the proposed example, ***x***_*n*_ represents the out-passenger-flows at the subway station “Window of World” during time window 8:45 a.m.–9:00 a.m. of the *n*th day in the training dataset, and ***y***_*n*_ represents the out-passenger-flows of the “Window of World” station during time window 9:00 a.m.–9:15 a.m. of the *n*th day in the training dataset.

#### Multilayer perceptron neural network model

The multilayer perceptron is a forward structure artificial neural network that maps a set of input vectors to a set of output vectors. An MLP consists of multiple layers, including an input layer, one or more hidden layers, and an output layer. Each layer of neurons is interconnected with the next layer of neurons. There is no connection between neurons in the same layer, and there is no cross-layer connection. For both the hidden layer and output layer, neurons have activation functions, whereas on the input layer, neurons only receive the input dataset and do not have activation functions. The learning process in neural networks involves adjusting the connection weights between neurons and the threshold of each functional neuron.

We considered a three-layer MLP network consisting of *d* input neurons, a hidden layer with *q* hidden neurons, and an output layer with *l* output neurons. The threshold of the *j*th neuron in the output layer is defined as *θ*_*j*_, and the threshold of the *h*th neuron in the hidden layer is defined as *γ*_*h*_. Connection weight *v*_*ih*_ represents the weight between the *i*th neuron in the input layer and the *h*th neuron in the hidden layer, whereas connection weight *w*_*hj*_ represents the weight between the *h*th neuron in the hidden layer and the *j*th neuron in the output layer. Therefore, each hidden neuron *h* firstly computes the net input $\alpha_{h}=\sum_{i=1}^{d}v_{ih}x_{i}$ and generates an output *b*_*h*_. Each output neuron *j* uses the outputs of the hidden layer as inputs $\beta_{j}=\sum_{h=1}^{q}w_{hj}b_{h}$.

For a single training sample (***x***_*k*_,***y***_*k*_), ${\hat{y}}_{k}=({\hat{y}}_{1}^{k},{\hat{y}}_{2}^{k},\ldots ,{\hat{y}}_{l}^{k})$ is the output of the neural network, that is ${\hat{y}}_{j}^{k}=f(\beta_{j}-\theta_{j})$, where *f*(∙) is the activation function, and the rectified linear unit function *f*(*x*) = max(0,*x*) is used here as the activation function. Therefore, the mean square error of the network is

$$E_{k}=\frac{1}{2}\sum_{j=1}^{l}{\left({\hat{y}}_{j}^{k}-y_{j}^{k}\right)}^{2}.$$ (1)

The update of any parameter *v* is defined as *v* ← *v* + Δ*v*. The training process of the MLP with backpropagation is as follows.

Step 1: Input the training dataset *D* = {(***x***_1_,***y***_1_),(***x***_2_,***y***_2_),…,(***x***_*n*_,***y***_*n*_)},***x***_*n*_ ∈ *R*^*d*^,***y***_*n*_ ∈ *R*^*l*^ and determine the activation function. In this paper, the number of hidden neurons *q* was set to 100, the tolerance for stopping criterion is set to 0.0001(i.e. value of (1) is smaller than 0.0001), and the maximum number of iterations is 200.

Step 2: All connection weights and thresholds in the neural network are initialized randomly in the range of (0, 1).

Step 3: For (***x***_*k*_,***y***_*k*_), according to current parameters and function ${\hat{y}}_{j}^{k}=f(\beta_{j}-\theta_{j})$, calculate the value of ${\hat{y}}_{k}$. The mean square error of the network is computed as $E_{k}=\frac{1}{2}\sum_{j=1}^{l}{\left({\hat{y}}_{j}^{k}-y_{j}^{k}\right)}^{2}$.

Step 4: Update the connection weights *w*_*hj*_ and *v*_*ih*_ and the thresholds *θ*_*j*_ and *γ*_*h*_.

$$w_{hj}\leftarrow w_{hj}+{\Delta w}_{hj},$$ (2)

$$v_{ih}\leftarrow v_{ih}+\Delta v_{ih},$$ (3)

$$\theta_{j}{\leftarrow \theta }_{j}+\Delta \theta_{j},$$ (4)

$$\gamma_{h}\leftarrow \gamma_{h}+\Delta \gamma_{h}.$$ (5)

The error backpropagation (BP) algorithm based on the gradient descent strategy adjusts the parameters [46, 47].

Step 5: Repeat Steps 1–4 until the value of (1) satisfies the predefined tolerance for stopping criterion.

#### Support vector regression model

The kernel function *Φ* is used to map data into a high-dimensional feature space, such that the nonlinear fitting problem in the input space is transformed into a linear fitting problem in the high-dimensional feature space. Common kernel functions include linear kernel, polynomial kernel, gaussian kernel, Laplace kernel, and sigmoid kernel, where the nonlinear mapping function is *k*(***x***_*i*_,***x***_*j*_) = *Φ*(***x***_*i*_)^*T*^ ∙ *Φ*(***x***_*j*_). The goal of the support vector regression model is to find the partition hyperplane with the maximum margin. The partition hyperplane is represented by *f*(***x***) = ***w***^*T*^*Φ*(***x***) + *b*, where ***w*** ∈ *R*^*d*^ is the normal vector, and *b* is the displacement.

Suppose *ε* is the error bound between observation value *y* and predicted value *f*(***x***). With *f*(***x***) as the center, the epsilon-tube with a width of 2*ε* is established, and then the problem is formalized [46] as

$$\min_{w,b}\frac{1}{2}{\left\|w\right\|}^{2}+C\sum_{i=1}^{n}l_{\varepsilon }(f(x_{i})-y_{i}),$$ (6)

where *C* is the penalty coefficient, and

$$l_{\varepsilon }(z)=\left\{ \begin{array}{cc} 0, & if|z|\leq \varepsilon ;\\ |z|-\varepsilon , & otherwise. \end{array}\right.$$ (7)

In summary, the support vector regression model can be described as follows.

Step 1: Input training dataset *D* = {(***x***_1_,***y***_1_),(***x***_2_,***y***_2_),…,(***x***_*n*_,***y***_*n*_)},***x***_*n*_ ∈ *R*^*d*^,***y***_*n*_ ∈ *R*^*l*^ and select a kernel function *k*(***x***_*i*_,***x***_*j*_). In this paper, the linear kernel was chosen as the kernel function, the parameter *C* was set to 1, *ε* was set to 0.1, and the tolerance for stopping criterion is set to 0.001 (i.e. the value of (6) is smaller than 0.001).

Step 2: Search for the best solution ${\bar{\alpha }}^{\left(*\right)}=({\bar{\alpha }}_{1},{{\bar{\alpha }}_{1}}^{*},\ldots ,{\bar{\alpha }}_{l},{{\bar{\alpha }}_{l}}^{*})$ to

$$\min_{\alpha ,\alpha^{*}}\frac{1}{2}\sum_{i,j=1}^{n}\left(\alpha_{i}^{*}-\alpha_{i})(\alpha_{j}^{*}-\alpha_{j})k(x_{i},x_{j}\right)+\varepsilon \sum_{i=1}^{n}(\alpha_{i}+\alpha_{i}^{*})-y_{i}\sum_{i=1}^{n}\left(\alpha_{i}^{*}-\alpha_{i}\right)$$ (8)

$$s.t.\sum_{i=1}^{n}(\alpha_{i}-\alpha_{i}^{*})=0$$

$${0\leq \alpha }_{i},\alpha_{i}^{*}\leq C,\;i=1,2,\ldots ,n.$$

Step 3: Calculate parameter *b*. Select the positive subvector of $\bar{\alpha }\;({\bar{\alpha }}_{j}>0)$, or the positive sub vector of ${\bar{\alpha }}^{*}\;({{\bar{\alpha }}_{j}}^{*}>0)$, and calculate parameter

$$b=y_{j}-\sum_{i=1}^{n}({{\bar{\alpha }}_{i}}^{*}-{\bar{\alpha }}_{i})k(x_{i},x_{j})+\varepsilon ,{0<\bar{\alpha }}_{j}<C,$$

$$b=y_{j}-\sum_{i=1}^{n}({{\bar{\alpha }}_{i}}^{*}-{\bar{\alpha }}_{i})k(x_{i},x_{j})-\varepsilon ,0<{{\bar{\alpha }}_{j}}^{*}<C.$$ (9)

Step 4: Obtain the model

$$f(x)=\sum_{i=1}^{n}\left({\alpha_{i}^{*}-\alpha }_{i}\right)k(x,x_{i})+b.$$ (10)

### Gradient boosted regression trees model (GBRT)

The gradient boosted regression trees model (GBRT) is described as follows.

Step 1: Input the training dataset *D* = {(***x***_1_,***y***_1_),(***x***_2_,***y***_2_),…,(***x***_*n*_,***y***_*n*_)},***x***_*n*_ ∈ *R*^*d*^,***y***_*n*_ ∈ *R*^*l*^ and initialization function

$$f_{0}(x)={arg\;min}_{c}\sum_{i=1}^{n}L(y_{i},c).$$ (11)

The loss function is $L(y,f(x))=\frac{1}{2}{\left(y-f(x)\right)}^{2}$, where the constant value *c* minimizes the value of $\sum_{i=1}^{n}L(y_{i},c)$, namely, *c* is as close as possible to ***y***_*i*_. Here, *f*_0_(***x***) is a tree with only one node.

Step 2: The training dataset is used as input to iteratively build *M* trees, *M* was set to 100 in this paper.

(a) For the *m*th tree, *m* = 1,…,*M*, calculate the negative gradient of the loss function in the current model

$$r_{mi}=-[\frac{\partial L(y_{i},f(x_{i}))}{\partial f(x_{i})}{]}_{f(x)=f_{m-1}(x)}.$$ (12)

Then, use *r*_*mi*_ as an estimate of residuals, where *i* = 1,…,*n*, ∂ stands for the derivative, and *n* is sample size.

(b) Fit a regression tree for *r*_*mi*_ to obtain the leaf node regions *R*_*mj*_ of tree *m*, where *j* = 1,2,…,*J*, and *J* is the number of leaf nodes, which is not limited in the present study.

(c) For the leaf node region *R*_*mj*_, where *j* = 1,2,…,*J*, calculating the best fitting value *c*_*mj*_ to minimize the loss function *L*(***y***,*f*(***x***)).

$$c_{mj}={arg\;min}_{c}\sum_{x_{i}\in R_{mj}}L(y_{i},f_{m-1}(x_{i})+c).$$ (13)

Then, update

$$f_{m}(x)=f_{m-1}(x)+\sum_{j=1}^{J}c_{mj}I(x\in R_{mj}).$$ (14)

Step 3: The final prediction model is

$$\tilde{f}(x)=f_{M}(x)=\sum_{m=1}^{M}\sum_{j=1}^{J}c_{mj}I(x\in R_{mj}).$$ (15)

### Detecting anomalous passenger flow condition

The DBSCAN algorithm was used to identify anomalous passenger flows. We normalized in- or out-passenger-flow of a subway station *s* during each time window *t* of a day *N*(*s*,*t*) with the minimum and maximum values of *N*(*s*,*t*) observed in the same time window during the whole data collection period, and take it as the original data set *S*. In the DBSCAN algorithm, the maximum radius of neighborhood *ε* defines the eps-neighborhood of a data point *i* ∈ *S*, denoted by *N*_*ε*_(*i*) = {*j* ∈ *S*|*dist*(*i*,*j*) ≤ *ε*}, and MinPts determines the minimum number of data points within the eps-neighborhood. The Euclidean distance *dist*(*i*,*j*) = |*N*(*s*,*t*)^*j*^ − *N*(*s*,*t*)^*i*^| was used to locate the *ε* neighborhood of each data point *i*, and the typical parameter setting of MinPts = 4 was used. The maximum radius of neighborhood *ε* was set using the fourth distance (4-dist) probability [50]: the distance between a data point and its fourth nearest neighbor is denoted as the 4-dist. The probability distribution of 4-dist was fitted by an exponential function, and the 4-dist value at which the slope of the fitting curve equaled -1 was used as the parameter setting of *ε*.

**Fig 3:**
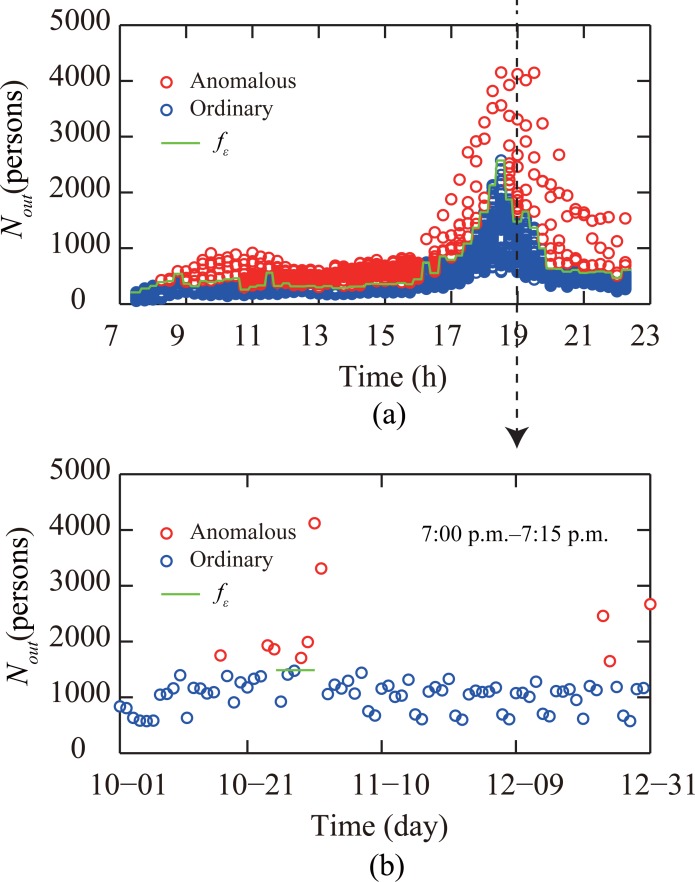
Anomalous passenger flow detection for “Window of World” subway station. (a) Out-passenger-flows of the “Window of World” subway station over the whole observation period. Anomalous flows and ordinary flows are discriminated by the green line *f*_*ε*_. (b) Red circles represent anomalous out-passenger-flow of the station during the time window 7:00 p.m.–7:15 p.m., while blue circles represent passenger flows under ordinary traffic conditions.

Passenger flows were classified using the DBSCAN algorithm: passenger flows larger than the maximum flow *f*_*ε*_ of the largest cluster were classified into the anomalous passenger flow (traffic) condition. Passenger flows smaller than or equal to *f*_*ε*_ were classified into the ordinary traffic condition. We use the out-passenger-flows *N*_*out*_(*s*,*t*) at the subway station “Window of World” during the time window 7:00 p.m.–7:15 p.m. as an example (Fig 3B). Here, *s* denotes the subway station “Window of World”, the target time window of the prediction is *t* = 76. The label of each cluster generated by the DBSCAN algorithm is denoted by the *label*(*r*), where 1 ≤ *r* ≤ *n*_*c*_,*n*_*c*_ is the total number of clusters. During time window *t* of the *i*th day, the out-passenger-flows at the studied station *s* is denoted as *label*(*N*_*out*_(*s*,*t*)^*i*^). When the label *label*(*N*_*out*_(*s*,*t*)^*i*^) is the same with the label of the largest cluster generated *label*(*r*)_*max*_, the threshold passenger flow *f*_*ε*_ is determined $f_{\varepsilon }=\max_{\mathrm{label}({N_{\mathrm{out}}(s,t)}^{i})={\mathrm{label}(r)}_{\max}}{\left(N_{out}(s,t\right)}^{i})$.

In Fig 3A out-passenger-flows *N*_*out*_(*s*,*t*) at “Window of World” station of the Shenzhen subway system are illustrated for every 15 min time windows. Using the DBSCAN algorithm, the threshold passenger flow *f*_*ε*_ for each 15 min time window was determined. Anomalous growth of passenger flows was observed on December 31, which was caused by the firework show at the plaza of the recreational park at “Window of World” [51]. For all subway stations, anomalous in-passenger-flows were found in 12.2% of time windows, whereas out-passenger-flow were found in 10.3% of time windows.

## Results

### Predicting dynamical passenger flows

Previous passenger flow prediction models have been seldom analyzed under anomalous traffic conditions, such as abrupt bursts of passenger flows in a particular subway station due to mass commercial or recreational events. Under anomalous traffic conditions, passenger demands may exceed the maximum capability that a subway station can provide; emergent managements are therefore required to protect the safety and order of subway transportation. In addition, under large crowd gatherings, subway service restrictions can be an important way to prevent passengers from flowing into the crowded area, hence avoiding dangerous crowding situations [52]. Therefore, predicting passenger flows under anomalous traffic conditions is even more important than predicting flow under ordinary conditions.

Three typical indexes, mean absolute percentage error (MAPE), variance of absolute percentage error (VAPE), and root mean square error (RMSE) were used to evaluate the accuracy of prediction:

$$MAPE=\frac{1}{n}\sum_{i=1}^{n}|\frac{y_{i}-{\hat{y}}_{i}}{y_{i}}|\times 100\%,$$ (16)

$$VAPE=Var\left(\frac{\left|y_{i}-{\hat{y}}_{i}\right|}{y_{i}}\right)\times 100\%,$$ (17)

$$RMSE=\sqrt{\frac{1}{n}\sum_{i=1}^{n}{{(\hat{y}}_{i}-y_{i})}^{2}},$$ (18)

where *y* = {*y*_1_,*y*_2_,…,*y*_*i*_,…*y*_*n*_} is the sequence of the observation values, $\hat{y}=\{{\hat{y}}_{1},{\hat{y}}_{2},\ldots ,{\hat{y}}_{i},\ldots {\hat{y}}_{n}\}$ is the sequence of the prediction values, and *n* is the number of observation values.

**Fig 4:**
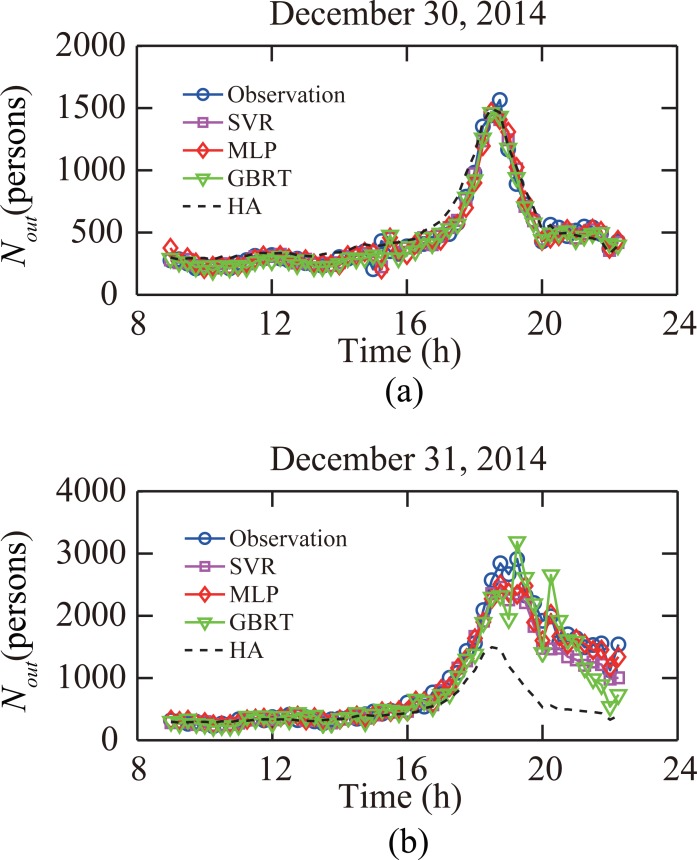
Predictive results versus ground-truth data for subway station “Window of World” when *n*_*step*_ = 1. (a) Results for a typical weekday. (b) Results for a day when mass events occurred near the station.

Fig 4 shows the prediction results and the ground-truth passenger flow observation at the subway station “Window of World” during a typical weekday (December 30, 2014) and a day when mass events occurred at the plaza near the subway station (December 31, 2014). The performances of four predictive models were similar in the ordinary traffic condition, and all models offered accurate prediction results. However, under the anomalous traffic condition, the HA model failed to capture the trend of abrupt growth of passenger flow as expected, and the prediction of the GBRT model had large fluctuations. Meanwhile, the SVR model and MLP model had a relatively good performance. The results shown in Fig 4 highlight the importance of selecting the proper model under different traffic conditions.

Table 1 shows the RMSE, MAPE, and VAPE values of predictive results of passenger flows at the “Window of World” station based on the SVR, MLP, GBRT and HA model. The prediction time is *n*_*step*_ = 1 time window ahead of the target time window.

**Table 1:** The average error of the four models when *n*_*step*_ = 1.

Method	December 30			December 31		
	RMSE	MAPE (%)	VAPE (%)	RMSE	MAPE (%)	VAPE (%)
SVR	56.22	8.83	1.30	209.10	12.98	0.60
MLP	68.91	11.50	1.39	145.58	10.33	0.44
GBRT	50.37	9.22	0.95	274.75	17.11	1.72
HA	86.72	19.20	2.23	718.70	29.12	6.72

### High-performance regions of different predictive models

**Fig 5:**
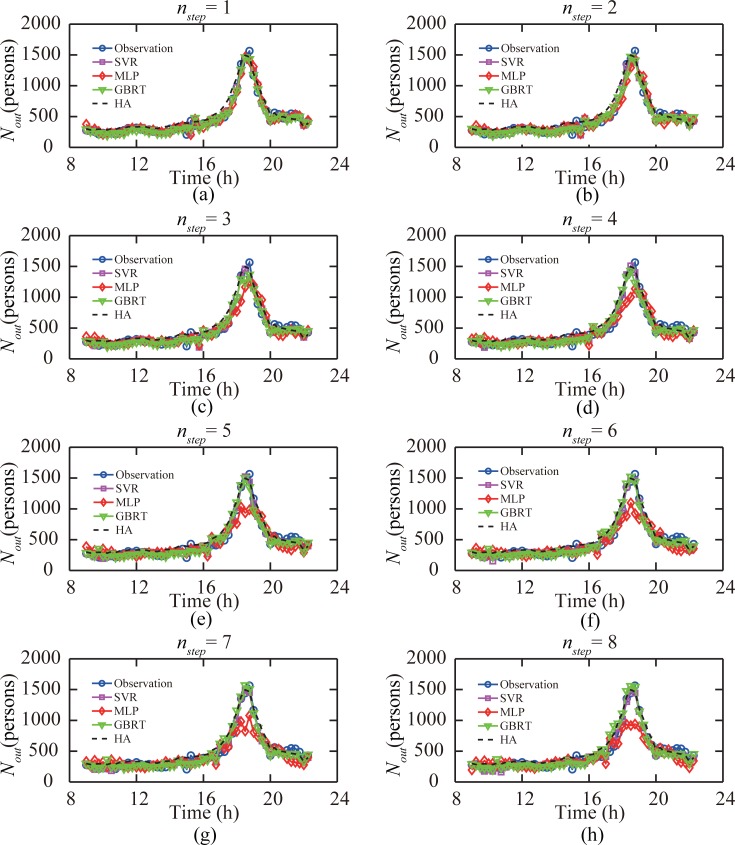
Performance of four models when predicting out-passenger-flow records in subway station “Window of World” on December 30, 2014. (a-h) When the prediction was made 1 to 8 time windows ahead of the target time window, respectively.

We analyzed the performances of four predictive models under different numbers of time windows *n*_*step*_ that a prediction is made before the target time window *n*_*target*_. When a larger *n*_*step*_ was set, the passenger flow prediction results could be obtained early; meanwhile, the accuracy of prediction decreased as more recent data were not used. Here, we explored the high-performance regions of different predictive models under ordinary and anomalous traffic conditions. Fig 5 shows the predicted passenger flows at the subway station “Window of World” on December 30, 2014. We found that under the ordinary condition, except for the MLP model, the predictive models performed well even when the passenger flow prediction was conducted 2 h before the target time window. The prediction accuracy of the MLP model began to decrease when *n*_*step*_ was larger than two time windows, indicating that under the ordinary traffic condition the MLP model only worked well for short-term (less than 30 min) prediction.

**Fig 6:**
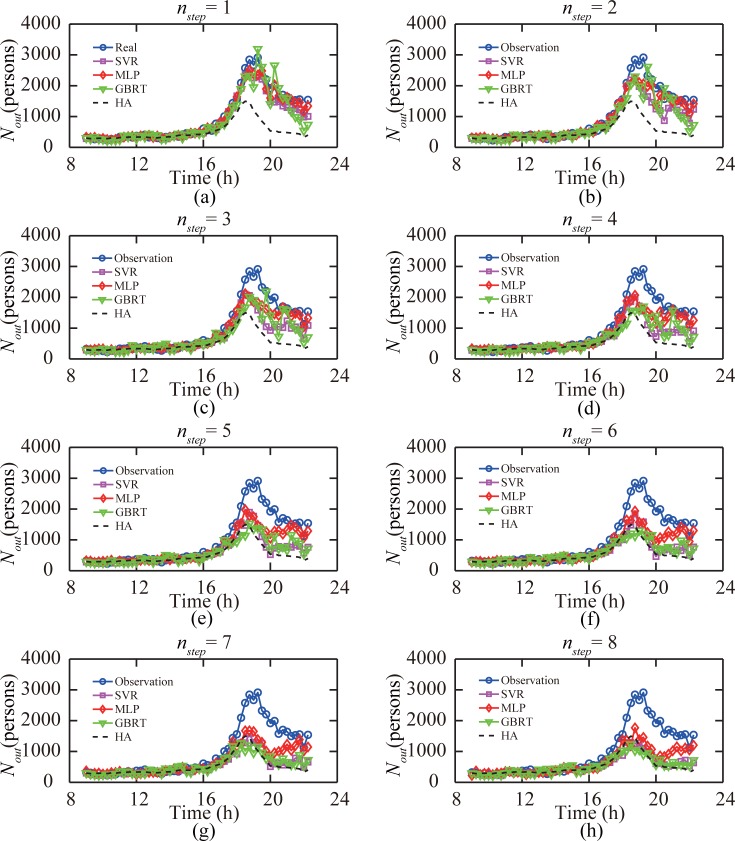
Performance of four models when predicting out-passenger-flow records in subway station “Window of World” on December 31, 2014. (a-h) When the prediction was made 1 to 8 time windows ahead of the target time window, respectively.

Fig 6 shows the predicted passenger flows of the subway station “Window of World” on December 31, 2014. In contrast to the results under the ordinary traffic condition, we found that under anomalous traffic condition, the MLP model performed the best. Given that the HA model is insensitive to the prediction time, the same predictive results were obtained for different numbers of time windows *n*_*step*_ that a prediction is made before the target time window, and the HA model could not capture the anomalous traffic condition at all. For all predictive models, the prediction accuracy was not acceptable when the target time window was four time windows (1 h) later than the prediction time. The predicted results of all models had a trend to approach historical average values when *n*_*step*_ ≥ 4.

Table 2 shows the RMSE, MAPE, and VAPE values of predictive results of passenger flows at the “Window of World” station based on the SVR, MLP, GBRT and HA model. The prediction was made 1 to 8 time windows ahead of the target time window, respectively.

**Table 2:** The average error of the four models.

RMSE								
	December 30				December 31			
*n*_*step*_	SVR	MLP	GBRT	HA	SVR	MLP	GBRT	HA
1	56.22	68.91	50.37	86.72	209.10	145.58	274.75	718.70
2	56.68	88.19	58.99	86.72	335.46	216.29	346.33	718.70
3	60.22	110.36	61.43	86.72	434.83	285.31	413.02	718.70
4	57.31	131.77	74.33	86.72	510.15	346.55	507.99	718.70
5	54.90	144.90	71.67	86.72	576.58	403.94	588.75	718.70
6	53.65	151.60	68.89	86.72	612.60	449.57	649.52	718.70
7	55.40	154.21	72.24	86.72	649.76	490.85	689.43	718.70
8	56.16	159.15	85.71	86.72	691.83	536.35	726.10	718.70
MAPE(%)								
	December 30				December 31			
*n*_*step*_	SVR	MLP	GBRT	HA	SVR	MLP	GBRT	HA
1	8.83	11.50	9.22	19.20	12.98	10.33	17.11	29.12
2	10.09	13.50	10.22	19.20	16.80	11.73	19.59	29.12
3	9.98	15.85	10.26	19.20	20.30	14.08	23.90	29.12
4	11.08	18.48	11.96	19.20	23.76	16.53	25.77	29.12
5	10.91	19.14	12.17	19.20	27.06	18.56	28.51	29.12
6	10.72	19.83	12.37	19.20	28.70	21.94	30.08	29.12
7	11.67	20.06	13.08	19.20	28.90	22.80	31.30	29.12
8	11.17	21.03	12.81	19.20	30.15	24.18	33.66	29.12
VAPE(%)								
	December 30				December 31			
*n*_*step*_	SVR	MLP	GBRT	HA	SVR	MLP	GBRT	HA
1	1.30	1.39	0.95	2.23	0.60	0.44	1.72	6.72
2	1.42	1.54	0.90	2.23	1.41	0.91	2.06	6.72
3	1.02	1.29	0.81	2.23	1.91	0.94	2.62	6.72
4	0.97	1.87	0.91	2.23	2.93	1.38	3.21	6.72
5	0.82	1.47	1.30	2.23	3.43	1.75	3.20	6.72
6	0.91	1.87	1.23	2.23	3.93	1.83	4.15	6.72
7	0.89	2.07	1.51	2.23	4.38	1.96	4.42	6.72
8	0.96	2.34	1.95	2.23	5.05	2.69	4.74	6.72

**Fig 7:**
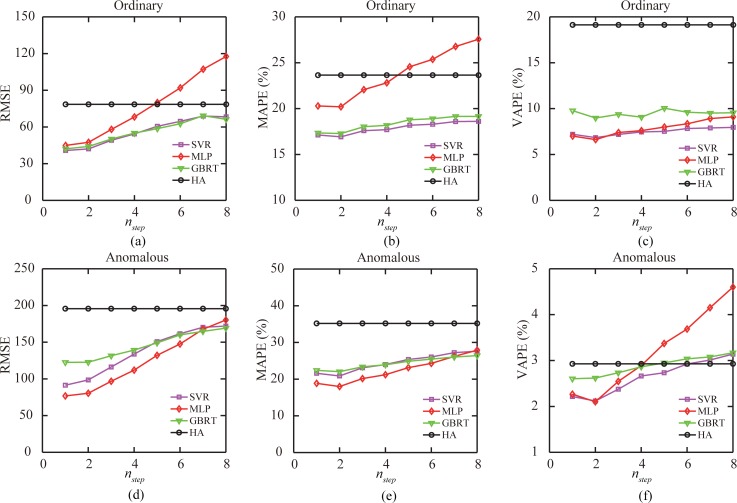
Performance analysis of the predictive models. (a), (b), (c) Performance of the four predictive models under the ordinary out-passenger flow condition for all stations during the whole test period. (d), (e), (f) Same as (a), (b), and (c), but for the results under the anomalous out-passenger flow condition.

We summarized the performance of the four predictive models in Fig 7. Under the ordinary traffic condition, the prediction errors of the SVR model, MLP model, and GBRT model all increased with the increase of the number of time windows *n*_*step*_ between the prediction time and target time windows. In particular, the RMSE and MAPE values of the prediction results of the MLP model increased much faster than for the SVR and GBRT models. When prediction was made *n*_*step*_ > 5 time windows earlier than the target time window, the MLP model had even worse performance than the HA model. The minimum MAPE = 16.9% was generated by the SVR model when *n*_*step*_ = 2, implying that the most recent data may be not the best data input. Furthermore, the GBRT model had a larger VAPE value than the MLP and SVR models. All the results taken together, the SVR model performed best in the ordinary traffic condition.

When a larger *n*_*step*_ was set (the prediction time (*t*) is earlier than the target time window *t*_*target*_, prediction errors of the SVR model, MLP model, and GBRT model increased faster under the anomalous traffic condition than under the ordinary traffic condition. The RMSE value and MAPE value of the prediction results of the MLP model, SVR model, and GBRT model were similar, but for most *n*_*step*_ settings the MLP model was slightly better. The minimum MAPE = 18.0% was generated by the MLP model when *n*_*step*_ = 2, also implying that the most recent data may be not the best data input. The GBRT model had a larger VAPE value when *n*_*step*_ was small, which increased slowly with increasing *n*_*step*_; meanwhile, the VAPE value for the prediction of the MLP model was small when *n*_*step*_ ≤ 2, but had faster growth afterward. Ultimately, the MLP model performed best in the anomalous traffic condition. Table 3 and Table 4 shows the RMSE, MAPE, and VAPE values of the four models under different *n*_*step*_ settings for in-passenger-flow and out-passenger-flow predictions.

**Table 3:** The average error of all in-passenger-flow of all stations.

RMSE								
	Ordinary				Anomalous			
*n*_*step*_	SVR	MLP	GBRT	HA	SVR	MLP	GBRT	HA
1	32.57	34.27	37.34	87.16	65.71	58.17	86.38	162.09
2	41.15	44.24	43.32	87.16	86.69	73.54	92.37	162.09
3	51.91	56.98	51.11	87.16	98.04	89.02	98.77	162.09
4	64.68	70.85	58.38	87.16	105.96	102.05	106.85	162.09
5	72.32	82.74	64.39	87.16	115.83	115.81	111.66	162.09
6	75.65	92.17	68.65	87.16	127.21	130.67	118.09	162.09
7	78.04	98.19	69.72	87.16	128.26	141.21	124.02	162.09
8	79.88	102.37	70.66	87.16	126.41	148.13	127.95	162.09
MAPE(%)								
	Ordinary				Anomalous			
*n*_*step*_	SVR	MLP	GBRT	HA	SVR	MLP	GBRT	HA
1	15.16	17.05	15.62	24.22	17.12	14.78	18.18	34.21
2	15.75	18.68	16.35	24.22	18.83	16.54	19.73	34.21
3	16.48	20.41	17.05	24.22	20.35	18.14	20.56	34.21
4	17.09	22.01	17.72	24.22	21.79	20.05	21.54	34.21
5	17.50	23.27	18.02	24.22	23.17	22.17	22.39	34.21
6	17.88	24.60	18.46	24.22	24.35	24.22	23.02	34.21
7	18.11	25.53	18.68	24.22	24.67	26.08	23.52	34.21
8	18.32	26.43	18.80	24.22	25.32	28.10	23.91	34.21
VAPE(%)								
	Ordinary				Anomalous			
*n*_*step*_	SVR	MLP	GBRT	HA	SVR	MLP	GBRT	HA
1	5.66	6.70	7.46	13.38	2.13	2.01	2.17	2.27
2	5.87	6.86	7.91	13.38	2.25	2.34	2.40	2.27
3	7.01	8.69	9.24	13.38	2.39	2.48	2.39	2.27
4	8.09	9.98	10.39	13.38	2.65	3.08	2.60	2.27
5	8.58	9.50	10.24	13.38	3.21	4.14	2.77	2.27
6	9.68	11.27	11.48	13.38	3.53	5.28	2.80	2.27
7	9.34	10.61	11.23	13.38	3.42	6.34	2.86	2.27
8	9.37	10.97	10.95	13.38	3.67	8.11	2.93	2.27

**Table 4:** The average error of all Out -passenger-flow of all stations.

RMSE								
	Ordinary				Anomalous			
*n*_*step*_	SVR	MLP	GBRT	HA	SVR	MLP	GBRT	HA
1	40.76	44.95	42.15	78.53	91.41	76.78	122.53	195.62
2	42.09	47.47	44.09	78.53	98.56	80.46	122.72	195.62
3	49.14	58.06	50.20	78.53	116.20	96.77	131.51	195.62
4	54.38	68.22	54.99	78.53	133.71	111.96	139.23	195.62
5	60.54	80.00	58.68	78.53	150.79	132.11	149.09	195.62
6	64.63	91.99	62.68	78.53	161.55	147.50	159.90	195.62
7	68.90	107.22	69.25	78.53	170.30	167.59	164.74	195.62
8	68.33	117.66	66.26	78.53	171.93	180.23	169.14	195.62
MAPE(%)								
	Ordinary				Anomalous			
*n*_*step*_	SVR	MLP	GBRT	HA	SVR	MLP	GBRT	HA
1	17.12	20.29	17.35	23.65	21.60	18.86	22.40	35.19
2	16.92	20.20	17.28	23.65	20.87	17.99	22.03	35.19
3	17.59	22.07	18.02	23.65	23.10	20.16	23.36	35.19
4	17.70	22.81	18.17	23.65	23.97	21.20	23.91	35.19
5	18.19	24.56	18.79	23.65	25.37	23.14	24.87	35.19
6	18.30	25.37	18.90	23.65	26.04	24.29	25.47	35.19
7	18.59	26.77	19.15	23.65	27.22	26.20	26.03	35.19
8	18.61	27.56	19.15	23.65	27.60	27.89	26.40	35.19
VAPE(%)								
	Ordinary				Anomalous			
*n*_*step*_	SVR	MLP	GBRT	HA	SVR	MLP	GBRT	HA
1	7.21	7.01	9.78	19.13	2.21	2.27	2.60	2.93
2	6.84	6.63	8.98	19.13	2.12	2.10	2.62	2.93
3	7.19	7.40	9.38	19.13	2.37	2.55	2.73	2.93
4	7.46	7.60	9.07	19.13	2.66	2.89	2.86	2.93
5	7.52	8.00	10.05	19.13	2.73	3.37	2.95	2.93
6	7.83	8.35	9.61	19.13	2.92	3.69	3.04	2.93
7	7.89	8.92	9.51	19.13	3.01	4.15	3.08	2.93
8	7.96	9.10	9.55	19.13	3.14	4.60	3.17	2.93

Given that all validations were made for all subway stations (118 in total) of the Shenzhen subway network, the average MAPE and RMSE values were enhanced by the majority of low-passenger-flow stations. If we concentrated on subway stations with the top 25% average passenger flows, the minimum MAPE = 11.1% was generated by the SVR model (and GBRT model) when *n*_*step*_ = 2 for the ordinary traffic condition; meanwhile, the minimum MAPE = 12.3% was generated by the MLP model when *n*_*step*_ = 2 for the anomalous traffic condition. This result indicates that the inherent pattern of passenger flows at a subway station prominently determines the prediction accuracy. In general, passenger flows of large-flow stations are more predictable than passenger flows of low-flow stations. In addition, for a specific group of subway stations, the best model may be different from the model obtained for all subway stations. In practice, more detailed model selection strategies can be implemented to different subgroups of subway stations. Table 5 and Table 6 describe details of RMSE, MAPE and VAPE values of the four models when *n*_*step*_ = 2.

**Table 5:** The average error of all in-passenger-flow (*n*_*step*_ = 2).

Method	TOP 25%			ALL		
	RMSE	MAPE (%)	VAPE (%)	RMSE	MAPE (%)	VAPE (%)
SVR	82.01	10.51	1.18	50.05	17.29	6.41
MLP	80.73	12.02	1.28	51.41	19.99	6.21
GBRT	87.22	10.90	1.74	56.18	17.72	8.41
HA	174.14	22.61	9.12	95.56	24.73	17.73

**Table 6:** The average error of all Out -passenger-flow (*n*_*step*_ = 2).

Method	TOP 25%			ALL		
	RMSE	MAPE (%)	VAPE (%)	RMSE	MAPE (%)	VAPE (%)
SVR	81.19	11.48	1.20	48.32	16.10	5.48
MLP	83.15	13.40	1.48	48.35	18.44	6.36
GBRT	94.29	11.58	1.46	51.09	16.73	7.31
HA	162.82	20.15	4.35	98.25	25.34	12.24

## Conclusions

An effective and reliable passenger flow prediction model can be beneficial to the management of transportation systems, such as operation planning, revenue planning, and facility improvement. In this paper, we generated four models to predict passenger flow in each station of the Shenzhen subway system. We investigated how long in advance passenger flows could be accurately predicted. Under ordinary traffic condition, acceptable results can be obtained even 2 h in advance, while under anomalous traffic condition, the prediction accuracy of all predictive models was not acceptable when prediction was made 1 h in advance. Li *et al*. [53] compared detrending models and multi-regime models trying to find appropriate traffic prediction models in practices. Our finding highlights the importance of selecting proper models, SVR model and MLP model respectively performed best in ordinary and anomalous traffic conditions. Our finding also highlights that compared with the selection of models, inherent patterns of passenger flows are more prominently influencing the accuracy of prediction. According to the analysis and results of the present study, when passenger flows are relatively stable, SVR prediction model is suggested. When passenger flows show anomalous patterns, the MLP prediction model can achieve more reliable prediction results. In addition, how long the prediction is made in advance of the target time window should also be considered. As the time window ahead of the target time window *n*_*step*_ increases, the prediction error increases. The prediction errors of the three types of knowledge discovery models gradually approach the prediction error of the simple HA model. Hence, in the condition that *n*_*step*_ is large, the simple HA model can be a good option given its low computation cost. We think our results can offer useful information for the management of public transportation, which includes adjusting operating frequency and alleviating passenger congestion.

Finally, we would like to discuss the limitations of this study, and future work. First, the four predictive models were used in their most basic forms and we did not cover all existing models for traffic prediction. Variants of these fundamental models could further improve the accuracy of predictions. Next, better classifications of traffic conditions or subway stations could further improve the prediction accuracy, and are worthy of future work. Finally, transportation information on social media websites is usually prior to the emergence of actual mobility, and therefore incorporating this kind of information with traditional urban transportation data is definitely an interesting future research direction [54, 55].

## Supporting information

S1 File The minimal dataset to replicate this study. (CSV)
